# Critical Issues in the Development of Health Information Systems in Supporting Environmental Health: A Case Study of Ciguatera

**DOI:** 10.1289/ehp.1002575

**Published:** 2010-12-16

**Authors:** Sarah Goater, Bonnie Derne, Philip Weinstein

**Affiliations:** School of Population Health, University of Queensland, Herston, Queensland, Australia

**Keywords:** ciguatera, climate change, ecosystem health, environmental health, health information systems

## Abstract

**Background:**

Emerging environmental pressures resulting from climate change and globalization challenge the capacity of health information systems (HIS) in the Pacific to inform future policy and public health interventions. Ciguatera, a globally common marine food-borne illness, is used here to illustrate specific HIS challenges in the Pacific and how these might be overcome proactively to meet the changing surveillance needs resulting from environmental change.

**Objectives:**

We review and highlight inefficiencies in the reactive nature of existing HIS in the Pacific to collect, collate, and communicate ciguatera fish poisoning data currently used to inform public health intervention. Further, we review the capacity of existing HIS to respond to new data needs associated with shifts in ciguatera disease burden likely to result from coral reef habitat disruption.

**Discussion:**

Improved knowledge on the ecological drivers of ciguatera prevalence at local and regional levels is needed, combined with enhanced surveillance techniques and data management systems, to capture environmental drivers as well as health outcomes data.

**Conclusions:**

The capacity of public HIS to detect and prevent future outbreaks is largely dependent on the future development of governance strategies that promote proactive surveillance and health action. Accordingly, we present an innovative framework from which to stimulate scientific debate on how this might be achieved by using existing larger scale data sets and multidisciplinary collaborations.

The capacity of Pacific Island nations to provide fish that are safe for consumption and trade will be challenged in the face of climate change. Although climatic drivers of coral reef disturbance are beyond our immediate control, their impact on public health could be minimized if an adaptive governance strategy were established to support health policy making, regulation, and coral reef and fish stock management. Health information systems (HIS) in the Pacific are designed to inform policy and public health intervention; however, their performance is being challenged by emerging problems such as environmental change (Goater et al. 2011). By way of example, we use the case study of ciguatera fish poisoning (referred to here as ciguatera), which is common throughout intertropical regions where coral reef systems occur (Lehane et al. 2000). Pacific island countries and territories (PICTs) rely heavily on coral reef fish stocks for food, export, and tourism because they provide a cheap, readily accessible natural resource for exploitation (Madin et al. 2008). Population growth, urbanization, pollution, and global pressures to meet industry and tourism demands are all forces shaping society and the environment in PICT areas (Gatti et al. 2008). The surrounding warm waters support a large percentage of the world’s coral reefs and are subject to cyclic or episodic changes caused by natural and anthropogenic events. With ecological disruption likely a key trigger for ciguatera outbreaks, the cumulative effects of globalization and climate change render ciguatera a priority health issue not only within the Pacific but potentially also on a global scale (Madin et al. 2008). Here, we investigated health surveillance data sets that are available to capture the disease burden of ciguatera in the Pacific and the reporting systems that are in place. We also assessed how these data are managed, analyzed, and disseminated to inform policy and public health interventions. Lastly, we have proposed an alternative approach to existing HIS, within a three-phase conceptual framework to stimulate debate over an optimal risk return for HIS and ciguatera in the Pacific.

## Ciguatera

**Figure 1:**
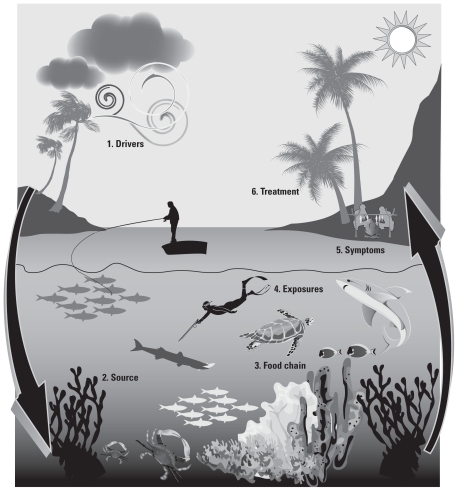
The ecology of ciguatera fish poisoning (adapted from Laurent et al. 2005). (*1*) Environmental factors such as cyclones, tsunamis, coral bleaching, reef blasting, and overfishing are all considered likely drivers of coral reef disruption linked to ciguatoxic microalgae *Gambierdiscus* spp. (*2*) Disruptions to the reef environment are thought to create an imbalance in the ecosystem, which increases the distribution of ciguatoxic microalgae throughout the reef and the likelihood of toxins entering the food chain. (*3*) Ciguatoxins enter the food chain via grazing herbivorous fish, which are in turn eaten by carnivorous fish, passing the toxin up through the food chain in a more concentrated form. (*4*) Eating toxic fish from any part of the food chain can poison humans, with more severe cases generally caused by consuming larger predatory fish, although this is not universally the case. (*5*) Signs occur between 2 and 12 hr after consuming toxic fish and include nausea, vomiting, diarrhea, slow pulse with normal temperature, and sweating. Other symptoms include numb or prickly sensations around lips, nose, hands, feet, and skin; temperature sensation reversal; muscle and joint aches; headaches; tiredness; shivering; and itchiness. (*6*) No treatment is available for ciguatera, only remedies to relieve discomfort or pain, comprising prescriptions and traditional practices.

Ciguatera is a food-borne illness in tropical regions that occurs when people eat marine fish that have been contaminated by natural toxins (Bagnis et al. 1980). Globally, ciguatera affects between 25,000 (Lewis and Sellin 1992) and 500,000 (Stinn et al. 1998) people per year (Figure 1). The potent neurotoxins responsible for ciguatera are predominantly ciguatoxins (Swift and Swift 1993). These toxins are odorless, tasteless, colorless, heat stable, and lipid soluble, remaining active after cooking, freezing, or smoking (Madin et al. 2008). The syndrome is characterized by a wide array of generalized and specific gastrointestinal and neurological symptoms (Gillespie et al. 1986) that may persist for months or several years after initial poisoning (Lewis 2006). There is no single remedy for the treatment of ciguatera, nor is there a reliable rapid bioassay for detection (Lewis 2006).

### Exposure pathways

The main causative organism of ciguatera is the unicellular dinoflagellate *Gambierdiscus toxicus* (Adachi and Fukuyo 1979) that lives upon common, larger reef-colonizing species of red, green, and brown algae (Lehane et al. 2000). Toxins enter the food chain when herbivorous fishes consume *G. toxicus* while grazing on these larger algae (Bagnis 1981; Bagnis et al. 1980; Yasumoto et al. 1977) (Figure 1). The toxins accumulate in the tissues of the fishes (Legrande et al. 1992) and bioaccumulate in higher predator species of the food chain—with humans at the apex of the trophic pyramid, experiencing the full effects of bioaccumulation. It is important to note that not all *Gambierdiscus* populations are toxic, not all fish species at a given location will accumulate toxins, and not all affected fish accumulate toxins to levels that are hazardous to human health (Parsons et al. 2010).

More than 300 fish species have been linked with the incidence of ciguatera among humans, with carnivorous fish including barracuda (*Sphyraenidae* spp.), parrotfish (*Scaridae* spp.), moray eels (*Muraenidae* spp.), Spanish mackerel (*Scombridae* spp.), and sea perch and snapper (*Lutjanidae* spp) possibly the most common sources of the toxins (Lehane et al. 2000). However, if *G. toxicus* is present and producing toxin in a given locality, all species in the local food webs may contain some level of toxin, for example, invertebrates including echinoderms such as sea urchins (holothurians) and crustaceans (lobster, crab) that graze upon reef-colonizing algae (Angibaud et al. 2000). Furthermore, consumption of predatory marine mammals and sea birds, or poultry and pigs fed on contaminated fishcakes, may also provide exposure pathways for humans (Angibaud et al. 2000).

### Intrinsic links to reef ecosystem health

**Figure 2:**
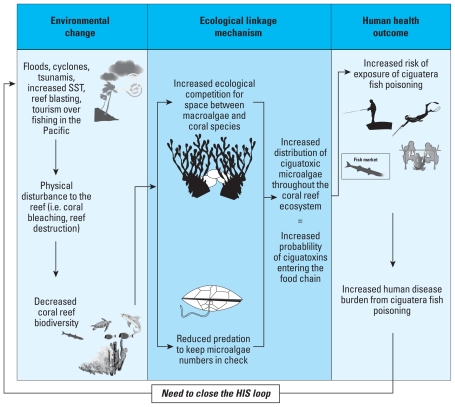
Linking environmental and human health outcome data to close the HIS loop. SST, sea surface temperature. The equal sign (=) indicates that increased distribution leads to increased probability.

Most publications on ciguatera have focused on clinical and epidemiological aspects (Chateau-Degat et al. 2005) rather than elucidating the interactions among the toxins, the algal hosts, and the ecological environment. Yet the incidence of ciguatera likely reflects the failure of coral reef ecosystems to acclimatize to the cumulative effect of changing environmental conditions (Figure 2). There are numerous hypotheses to explain the seasonality of toxin production (Chateau-Degat et al. 2005; Chinain et al. 1999), for example, sea surface temperature (Chateau-Degat et al. 2005; Chinain et al. 1999; Hales et al. 1999), *G. toxicus* growth (Bagnis 1981; Bomber et al. 1988; Chateau-Degat et al. 2007; Cook et al. 2004; Yasumoto et al. 1980), coral bleaching events (Chinain et al. 1999), and reef damage caused by infrastructure development (Anderson et al. 1983; Bagnis et al. 1980; Hearnden et al. 2003; Ruff 1989), sunlight, nutrient levels, ocean currents, and winds (Bomber et al. 1988), and rainfall events (Anderson et al. 1983). It has also been suggest that outbreaks occur only where environmental conditions favor the growth of highly toxic genetic strains of *Gambierdiscus* (Bagnis et al. 1980).

Where ciguatera case data have been linked to major environmental disturbance events, lag times and estimated times of recovery to safe fish supplies vary considerably (Anderson et al. 1983; Bagnis 1981; Chinain et al. 1999; Ruff 1989). Such variability illustrates the value of comparing environmental parameters alongside detailed records of ciguatera incidence within a given region. However, it is widely acknowledged that in order to trigger effective public health action, such research relies heavily on reliable health surveillance data and timely analysis (Chiller et al. 2005; Dickey and Plakas 2010).

## HIS in the PICT Region

Health care in the PICTs is delivered through a national system of governance, but case history and outbreak data are often inconsistent and fragmented, and the use of this data in local decision making and program evaluation is mixed (Lum On et al. 2009). A broader regional picture can be gained from the World Health Organization (WHO) or the Secretariat of the Pacific Community (SPC), both of which obtain data from each country. Longer term routine surveillance, including census data, birth and death registers, multiple-indicator cluster surveys, demographic health surveys, nutritional surveys, WHO STEPwise surveys, and donor research (Mokdad and Lozano 2009), yield additional data, but because they are often collected by agencies other than the health department, they may not be included in health decision making. Further, outbreak data may be collected by disease control units rather than health service providers and may not then be incorporated into the HIS (Chiller et al. 2005; Finau 1994). The geographic isolation and the diversity of populations, cultures, economies, and politics of PICTs are additional challenges to the consistent delivery of health systems across the Pacific region—communication and integration across these boundaries is not guaranteed (Chiller et al. 2005; Finau 1994).

### Ciguatera data collection

A major problem affecting routine data collection on ciguatera in the PICTs is inherent underreporting and misdiagnosis, because of the highly variable symptoms of poisoning and a prevalent cultural apathy toward seeking medical attention or reporting something that is considered part of island life (Chinain et al. 2010, Parsons et al. 2010). Recent advances in fish meal bioassay techniques can confirm diagnosis, whereas case history alone masks the true incidence of ciguatera. However, diagnostic laboratory capacity is limited in the Pacific and prevents the widespread use of bioassays (Chiller et al. 2005). To date, accurate documentation of ciguatera case histories has been confined mainly to French Polynesia, Australia, and Hawaii. Because these countries manage independent surveillance systems for ciguatera that are not part of the PICT regional HIS, we do not consider them in this article.

### Ciguatera data management and information products

Several attempts in the past 20 years have been made to capture the true health burden posed by ciguatera in the Pacific in an effort to inform policy and develop mechanisms of public health intervention. In 1988, the South Pacific Epidemiological and Health Information Service (SPEHIS) established the first formal regional mechanism of collecting fish poisoning records (Dalzell 1993). As a tool for informing policy and for preventing ciguatera, this system was severely limited, because information to distinguish ciguatera from other forms of fish poisoning, such as symptoms (tingling, temperature reversal) or the source of exposure (e.g., fish species, size, location of catch), was not collected. Further, although the system was initially successful, provision of data from the contributing countries was (and remains) voluntary. Consequently, few countries continue to report on an annual basis.

In 1990, the Integrated Coastal Fisheries Management Programme (ICFMaP 2010) established a separate database more specifically targeted at recording ciguatera. Data were collected via questionnaires from hospitals and clinics in SPC member countries. The purpose of these surveys was to collect and compile ongoing case history data. In addition to general information such as date and number of people affected, the questionnaire compiled information on the type of food, the ecological source of the food, food preservation techniques, the part of the fish consumed, and how the fish was prepared for eating. Further, a section on medical data detailed information on 18 different symptoms presented across the sample group. Although this program is an improvement on the SPEHIS database, an overview of the information collected suggests it is not representative of the region, because > 50% of documented case histories are from Tuvalu (Froese and Pauly 2000). Also, of the nonfish species identified as the organism ingested, 10% were not identified. As with the SPEHIS database the ICFMaP database ceased to be maintained once the program ceased in 1998.

Information from the ICFMaP database was later incorporated into a global environmental information system that provides a central repository on all fishes known to science (Froese and Pauly 2000). Within FishBase, a search tool for ciguatera extracts a list of all species that have been identified as causing the illness in published sources and the ICFMaP database. Each record includes information (when available) on the country where the poisoning was reported, a geographic indicator of the extent of poisoning (local, regional, or national); an indicator of frequency of occurrence (rare, occasional, or frequent); and a remarks section providing more precise information on locality. Data collated specifically from the ICFMaP database provide additional information on victim responses to the questionnaire. However, information on all nonfish marine organisms likely to be the causal organism has been omitted from FishBase.

A major limitation of FishBase to inform health services in Pacific nations and link to HIS is that case history data cannot be retrieved in a single file; instead data must be accessed via individual listings of the ≥ 300 species of fish that have been identified as causing ciguatera. These data therefore cannot be exported into a table that summarizes ciguatera case history data, nor can a geographic map of all ciguatera cases be readily produced. Further, the online version of FishBase does not provide access to case history data on questionnaire responses and must be purchased on a CD-ROM. The loss of information on nonfish species (e.g., invertebrates, marine mammals, birds) limits the ability to identify the pathway of ciguatoxin production within the food chain and forewarn of likely fish and human exposure. However, the SPC has committed to continue to provide information on new cases from the Pacific, with the FishBase web site (2010) also encouraging input from researchers around the world.

In 2006, the SPC and Institute of Research for Development coproduced a field guide on ciguatera that compiled and reviewed the current knowledge of the illness (Laurent et al. 2005). The field guide also proposed techniques to assess and reduce the risk of ciguatera, providing a strategic methodology for collecting detailed case history and background information, supplemented by the collection of algae from the source location to test for *G. toxicus*. The template questionnaire addresses many of the limitations highlighted with earlier surveillance efforts, and includes personal and contact information of the patient; the type of marine organism consumed, the source location of the habitat type, preservation method, the body part consumed, how the organism was cooked, and other consumer information (how many ate the meal, felt sick, were admitted to hospital); details of catch location, date, and time; a detailed list of symptoms (covering epidermal, ocular, muscular, excretory, fever and chills, etc.); medical data (pulse, blood pressure, pupils); and whether death resulted. The template for collecting background information is generally targeted at obtaining information on seafood poisoning history in family or friends of the person interviewed, including estimates of the number of times people fell ill, dates and times, seasonal observations, frequent symptoms, types of seafood causing illness, location of toxic seafood products, type of treatment administered, and knowledge of ciguatera. Despite the potential to considerably improve existing information within FishBase, no information is readily available on whether this guide has been implemented across the PICTs, and no such data were present in FishBase as of late 2009.

### How are ciguatera data analyzed and disseminated to inform health managers and services?

Exactly how data concerning food-borne illnesses, including ciguatera, are collectively captured and analyzed for translation into policy or community and regional scale health actions is unclear. Some public information brochures have been issued by the Ministry of Marine Resources in the Cook Islands warning that ciguatera outbreaks can be caused by environmental disturbances (Friedman et al. 2008; Hajkowicz 2006). Others recommend monitoring of the presence and abundance of *G. toxicus* and screening for contamination of endemic coral fish species, combined with public education after severe environmental disturbances such as by tsunami (Laurent et al. 2005). On balance, however, it appears that available food-borne surveillance data provide little reliable information upon which to base timely health interventions (Chiller et al. 2005). The absence of reliable data, and the general lack of use of available data across the PICTs, suggests that there is little value in continuing the collection of disease outcome data if effective means of prevention cannot be developed.

### Public health interventions for ciguatera

Although multidisciplinary research collaborations have improved our understanding of ciguatoxins and their transmission through the food chain (Dickey and Plakas 2010), few have resulted in proactive public health interventions for the PICTs. Bioassay techniques for detecting toxins in fish meals reduces diagnosis error, yet testing meals that have already been eaten is at the downstream end of the causality chain. Addressing the upstream end by restricting fish consumption and discouraging trade of select fishes based on size or region of origin (Sadovy 1999; Wong et al. 2005) may be effective in the short term for developed nations; however, it is not universally applicable in Pacific island nations, where livelihood and socioeconomic status is dependent on fish consumption. Longer-term challenges of fish consignment restrictions include the changing regional risk profile of ciguatera resulting from climatic, oceanographic or other environmental impacts on coral reef health (Dickey and Plakas 2010) and the increasing toxic potential of smaller specimens of restricted trade species, or smaller fish more generally, because of increased harmful algal bloom events (Lechuga-Devéze and Sierra-Beltrán 1995) and consequent ciguatoxin prevalence within the marine ecosystems (Dickey and Plakas 2010). The reactive nature of these research-derived interventions highlight a caveat in the adaptive capacity of health systems of Pacific nations to respond to future climate change impacts on coral reef habitats, and the dynamic nature of the disease burden of ciguatera.

## Strategies toward Developing Ciguatera Interventions: Linking Human and Environmental Information Systems

**Figure 3:**
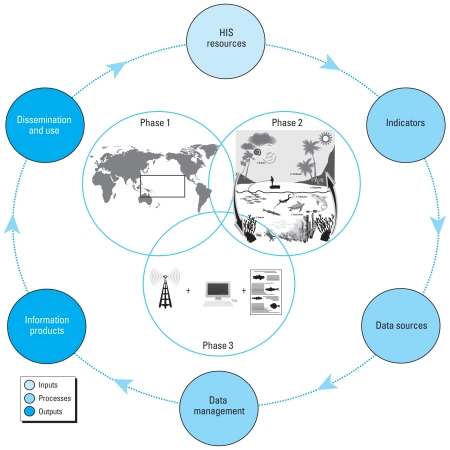
Three-phase approach to improving HIS for ciguatera in the Pacific. As presented, the health system loop (adapted from Health Metrics Network Framework; WHO 2009) depicts the dependence of government and donor research on reliable surveillance data to enable timely interpretation and dissemination of information products to trigger public health action (see “Strategies toward Developing Ciguatera Interventions: Linking Human and Environmental Information Systems”). In Phase 3, the drawing represents the use and integration of different technologies and methods of communication to best inform community-scale risk assessment. The plus signs (+) refer to the integration of these resources.

It is well recognized that beyond the biophysical environment, social, economic, and policy environments also affect the emergence of diseases in communities (Samet 2010). Thus, quantifying ciguatera-related risk requires an improved understanding of not only how human–environment relationships might change with varying environmental conditions but also how these relationships may change with different health policies and adaptive strategies. A perceived barrier hindering proactive risk assessment and prevention of ciguatera in PICT communities is the dearth of information on environmental drivers of ciguatoxin production and the associated public health risks (Dickey and Plakas 2010). We argue, conversely, that sufficient knowledge currently exists to commence developing a risk-based approach toward identifying and prioritizing at risk communities across the PICT region. Reflecting the sentiments of Chiller et al. (2005) and Chinain et al. (2010), we propose an upstream approach to improving HIS in the Pacific, through the development of progressively aggressive health interventions targeted at reducing the disease burden of ciguatera. The following three-phase risk prioritization framework is presented to stimulate scientific debate on how this might be achieved (Figure 3).

### Phase 1

Prioritize the use and integration of existing interdisciplinary data sets to provide a comprehensive knowledge base from which to focus HIS data collection, interpretation, and communication—without increasing demand on an already stretched health care system. Because of the vastness of the PICTs and the range of climatic and environmental conditions experienced, each community’s risk and vulnerability to water-related hazards will vary in both space and time (Parsons et al. 2010). By analyzing existing public health surveillance data with those hypothesized environmental drivers of ciguatera, potential exists to identify, classify, and rank those regions across the Pacific mostly likely to be exposed to ciguatoxic fish. Indeed, projecting ciguatera occurrence under varying environmental and climate scenarios will be surrounded by considerable uncertainty (e.g., the quality of public health surveillance data and the changing influence of future biological and socioeconomic adaptations on environment–health relationships are all contributing factors). Yet these uncertainties are not insurmountable if new statistical methods are embraced. The priority is to use those tools that can account for all known assumptions and uncertainties within a single theoretical environment, and that can be easily updated with new information and can prioritize where additional data collection are required to reduce uncertainty and strengthen ciguatera risk profiles [see Supplemental Material (doi:10.1289/ehp1002575)].

### Phase 2

Develop a series of “reef health” indicators that consider the interplay among the toxin, the host (marine and human), and the environment (marine, land use, and climate), to be used as triggers for proactive public health action. By categorizing and prioritizing at risk areas in phase 1, multidisciplinary research efforts can then be focused to improve knowledge on the biological and ecosystem stressors useful in establishing reef health indicators as part of an early warning system. New information that identifies and tracks toxins through all trophic levels will continually improve reef health indicators of likelihood and consequence of exposure to ciguatera. This information ought to be fed back into the hazard profiling tools used in phase 1. Such an approach would create a dynamic risk-mapping tool that can be used to generate public health notices to filter information back to community users.

### Phase 3

Close the HIS loop by feeding data and knowledge gained through phases 1 and 2 back into climate change projections to elucidate future health challenges and corresponding health policy strategies. In order to develop short-, medium-, and long-term strategies for improving community resilience to future ciguatera challenges, regional policy makers require simple tools that best inform community-scale risk assessment under varying climate and environmental change scenarios. Technology already exists, such as Geographical Information Systems (GIS), to develop integrated spatial tools to identify and rank PICT regions by likelihood of experiencing ciguatera in the context of climate and environmental change. Such tools could provide a platform from which to establish an adaptive governance strategy for making informed health policy by closing the HIS feedback loop and identify good HIS practices and use these as flagships for other at-risk communities. This approach to HIS would undoubtedly help policy makers avert or minimize risk arising from human–environment interactions caused by climate- and anthropogenic-induced ecosystem change.

By working collaboratively with external agencies to identify what information is required to best meet their health care needs, the PICT public health sector would be in a stronger position to develop proactive health policy and interventions. However, to do so, a dialogue among regional HIS services, health care practitioners, fisheries management, donor groups, and existing researchers needs to be initiated. Rekindling the momentum generated by SPC through workshops on ciguatera may prove an excellent vehicle for such a dialogue. Alternatively, existing programs such as the AusAid-funded HIS Knowledge Hub could provide a contact point for interested researchers. We would also welcome direct feedback from researchers who see merit in advancing and strengthening the conceptual framework proposed.

## Conclusions

The capacity of PICTs to cope with future challenges of ciguatera would be improved by using multidisciplinary resources oriented toward proactive surveillance and health action. We present our conceptual framework here to generate discussion about how such strategies might be achieved. We identify data sources and information systems that could be used in conjunction with existing knowledge to better inform HIS in PICT regions. We also propose a more targeted approach to the risk characterization of ciguatera zones to guide the collection, review, and refinement of information drawn from human and environmental data sets and so optimize risk return. The innovation of this approach is its transition from a management framework where health outcomes are largely reactive and dependent upon stretched health resources and capacity, to a proactive system subsidized by larger scale climate and environmental initiatives. The result—better understanding of the disease burden of ciguatera that is then used to prioritize or reallocate public health resources for the PICT members within an adaptive management framework.
